# Next-generation sequencing for genetic testing of familial colorectal cancer syndromes

**DOI:** 10.1186/s13053-015-0039-9

**Published:** 2015-08-21

**Authors:** Michele Simbolo, Andrea Mafficini, Marco Agostini, Corrado Pedrazzani, Chiara Bedin, Emanuele D. Urso, Donato Nitti, Giona Turri, Maria Scardoni, Matteo Fassan, Aldo Scarpa

**Affiliations:** 1 ARC-Net Research Centre, University and Hospital Trust of Verona, Verona, Italy; 2 Department of Surgery, Oncology and Gastroenterology, University of Padua, Padua, Italy; 3 Nano Inspired Biomedicine, Institute of Pediatric Research, Città della Speranza, Padua, Italy; 4 Department of Surgery, General Surgery A, University of Verona, Verona, Italy; 5 Department of Pathology and Diagnostics, University and Hospital Trust of Verona, Verona, Italy; 6 ARC-Net Research Centre, Department of Pathology & Diagnostics, University of Verona, Policlinico GB Rossi, Piazzale L.A. Scuro, 10, Verona, Italy

**Keywords:** Next generation sequencing, Colorectal adenocarcinoma, FAP, HNPCC

## Abstract

**Background:**

Genetic screening in families with high risk to develop colorectal cancer (CRC) prevents incurable disease and permits personalized therapeutic and follow-up strategies. The advancement of next-generation sequencing (NGS) technologies has revolutionized the throughput of DNA sequencing.

**Methods:**

A series of 16 probands for either familial adenomatous polyposis (FAP; 8 cases) or hereditary nonpolyposis colorectal cancer (HNPCC; 8 cases) were investigated for intragenic mutations in five CRC familial syndromes-associated genes (*APC*, *MUTYH*, *MLH1*, *MSH2*, *MSH6*) applying both a custom multigene Ion AmpliSeq NGS panel and conventional Sanger sequencing.

**Results:**

Fourteen pathogenic variants were detected in 13/16 FAP/HNPCC probands (81.3 %); one FAP proband presented two co-existing pathogenic variants, one in *APC* and one in *MUTYH*. Thirteen of these 14 pathogenic variants were detected by both NGS and Sanger, while one *MSH2* mutation (L280FfsX3) was identified only by Sanger sequencing. This is due to a limitation of the NGS approach in resolving sequences close or within homopolymeric stretches of DNA. To evaluate the performance of our NGS custom panel we assessed its capability to resolve the DNA sequences corresponding to 2225 pathogenic variants reported in the COSMIC database for *APC*, *MUTYH*, *MLH1*, *MSH2*, *MSH6*. Our NGS custom panel resolves the sequences where 2108 (94.7 %) of these variants occur. The remaining 117 mutations reside inside or in close proximity to homopolymer stretches; of these 27 (1.2 %) are imprecisely identified by the software but can be resolved by visual inspection of the region, while the remaining 90 variants (4.0 %) are blind spots. In summary, our custom panel would miss 4 % (90/2225) of pathogenic variants that would need a small set of Sanger sequencing reactions to be solved.

**Conclusions:**

The multiplex NGS approach has the advantage of analyzing multiple genes in multiple samples simultaneously, requiring only a reduced number of Sanger sequences to resolve homopolymeric DNA regions not adequately assessed by NGS. The implementation of NGS approaches in routine diagnostics of familial CRC is cost-effective and significantly reduces diagnostic turnaround times.

**Electronic supplementary material:**

The online version of this article (doi:10.1186/s13053-015-0039-9) contains supplementary material, which is available to authorized users.

## Background

Up to 30 % of colorectal cancers (CRC) have evidence of a familial component and about 5 % arise within well-characterized hereditary CRC syndromes [1–3]. The most frequent inherited CRC syndromes are: i) familial adenomatous polyposis (FAP), due to mutations in the adenomatous polyposis gene (*APC*); ii) *MUTYH*-associated polyposis (MAP), presenting mutations in the *MUTYH* gene; iii) hereditary nonpolyposis colorectal cancer (HNPCC), due to mutations in a DNA mismatch repair gene, most frequently *MLH1* or *MSH2* and rarely *MSH6* or *PMS2* [3–6].

The introduction of colorectal cancer screening programs has significantly decreased the occurrence of advanced CRC. However, large scale mutational screening in families with high incidence of cancer has been prevented by the high costs of Sanger DNA sequencing [7]. The introduction of next-generation sequencing (NGS) technologies has revolutionized the speed and throughput of DNA sequencing [8, 9], facilitating the genomic dissection of various types of human cancers, including CRC [10–12]. The capability of NGS technologies to simultaneously sequence multiple samples for multiple genes, starting from a limited amount of DNA [13–15], holds the promise to significantly reduce the costs of the analysis as well as the diagnostic response timing.

The purpose of this study was to compare a multigene NGS approach vs. Sanger sequencing for detection of intragenic mutations for diagnostic genetic testing of FAP and HNPCC.

## Methods

### Cases

A consecutive series of 16 blood samples obtained from 8 FAP and 8 HNPCC probands (11 females; mean age 42.4 ± 20.6 years, median 38.5 years) from the Clinical Surgery I at the University of Padua were used. All probands had a clinical history of familial CRC syndrome that had not been molecularly characterized. Each patient provided written informed consent for genetic testing.

### DNA extraction and quantification

DNA was purified using the QIAamp DNA Blood Mini Kit (Qiagen), and quantified using NanoDrop (Life Technologies) and Qubit (Life Technologies) platforms. DNA quality was further evaluated by PCR analysis using the BIOMED 2 PCR multiplex protocol with PCR products analyzed by DNA 1000 Assay (Life Technologies) on the Agilent 2100 Bioanalyzer on-chip electrophoresis (Agilent Technologies), as previously described [16].

### Deep Sequencing of Multiplex PCR Amplicons

An Ampliseq multigene custom panel was designed to explore all exons of *APC* (*n* = 16; NM_000038.5)*, MUTYH* (*n* = 16; NM_001128425.1)*, MLH1* (*n* = 17; NM_000249.3)*, MSH2* (*n* = 16; NM_000251.2)*, and MSH6* (*n* = 10; NM_000179.2) genes. The details of the target regions as produced by the AmpliSeq designer v2.2.1 are in Additional file 1: Table S1. Thirty nanograms of DNA were used for multiplex PCR amplification, followed by ligation of a specific barcode-sequence to each sample for identification. Emulsion PCR to construct the libraries of clonal sequences was performed with the Ion OneTouch™ OT2 System (Life Technologies). The quality of the obtained libraries was evaluated by the Agilent 2100 Bioanalyzer on-chip electrophoresis (Agilent Technologies) as previously described [16]. Sequencing of the libraries was performed on Personal Genome Machine (PGM, Life Technologies) using the Ion 318 Chip Kit v2. Four samples were processed in each emulsion PCR and sequencing. Data analysis, including alignment to the hg19 human reference genome and variant calling, was done using the Torrent Suite Software v3.6 (Life Technologies). Filtered variants were annotated using the SnpEff software v3.1. Alignments were visually verified with the Integrative Genomics Viewer (IGV) v2.2 (Broad Institute). Analysis of blind regions (where automated variant calling is hindered by sequencing errors due to homopolymers or amplification artifacts) was executed as follows: the COSMIC database of SNPs and small INDELs was converted to a Hotspots file and used to guide variant calling. In this way, the variant caller is forced to analyze a given hotspot coordinate; if there is no mutation, the software outputs that the position is “reference”; otherwise it outputs the mutation detected. If there are problems in the sequence at that position, the software outputs a “no call” value, explaining why variant calling failed (strand bias, quality of bases, noise in the sequence, low coverage). All the positions where a clear variant/reference status could not be called were further inspected by visual verification of the alignment file to ascertain whether the “no call’ status was due to artifacts or homopolymer misalignment.

### DNA Sanger Sequencing

All exons of *APC* and *MUTYH* for FAP probands and of *MLH1, MSH2* and *MSH6* for HNPCC probands were analyzed by conventional Sanger sequencing (primer sequences available upon request). PCR products were purified using Agencourt AMPure XP magnetic beads (Beckman Coulter) and labelled with BigDye® Terminator v3.1 (Applied Biosystems). Agencourt CleanSEQ magnetic beads (Beckman Coulter) were used for post-labeling DNA fragment purification, and sequence analysis was performed on the Applied Biosystems 3130xl Genetic Analyzer.

## Results

### Targeted next-generation sequencing

**Fig. 1:**
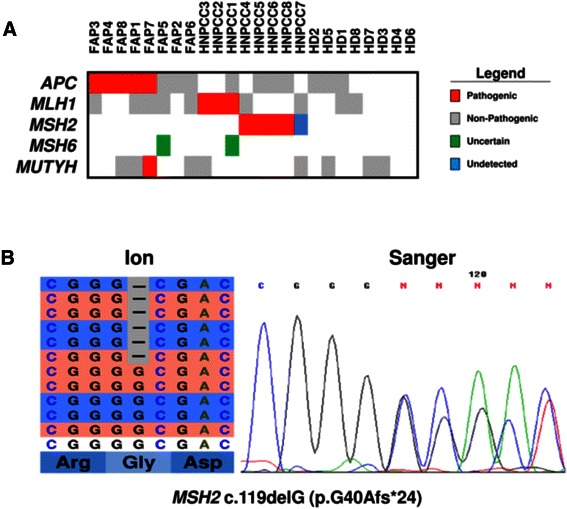
**a** Mutations detected at next generation sequencing with the Ion Ampliseq custom panel. **b** A representative example of Sanger sequencing validation of a mutation identified using next generation sequencing (sample HNPCC6). On the left is the representation of the results of next-generation sequencing where the reads are aligned to the reference genome as provided by the Integrative Genomics Viewer (IGV v.2.1, Broad Institute) software. On the right is the representation of the results of Sanger sequencing

The results of NGS target sequencing are shown in Table 1 and Fig. 1. DNA from all samples was successfully amplified in multiplex PCR for the 5 considered genes and an adequate library for NGS was obtained. The mean read length was 109.5 base pairs and a mean coverage of 1800x was achieved, with 97 % target bases covered more than 100x, and a minimum coverage of 20x in all cases.

**Table 1:** Mutations detected at next-generation and Sanger sequencing

Sample	*APC*	*MUTYH*	*MLH1*	*MSH2*	*MSH6*
FAP1	c.3433G > T p.E1145*				
FAP2					
FAP3	c.2805C > A p.Y935*				
FAP4	c.834 + 2 T > C				
FAP5					c.663A > C p.E221D^a^
FAP6					
FAP7	c.3920 T > A p.I1307K	c.536A > G p.Y179C			
FAP8	c.694C > T p.R232*				
HNPCC1			c.677G > A p.R226Q		c.998C > T p.T333I^a^
HNPCC2			c.432A > G p.T82A		
HNPCC3			c.1731G > A p.S577S		
HNPCC4				c.1386 + 1G > T	
HNPCC5				c.1216C > T p.R406*	
HNPCC6				c.119delG p.G40Afs*24	
HNPCC7				c.840_841delAT p.L280Ffs*3 ^b^	
HNPCC8				c.1046C > G p.P349R	

^a^ variants with uncertain pathogenic potential

^b^ variant detected only by Sanger sequencing

Among the 8 FAP probands, pathogenic mutations in *APC* gene were found in 5: three nonsense mutations, one missense mutation and one splice site alteration. Of interest, the c.3920 T > A (p.I1307K) *APC* missense alteration in proband FAP7 was associated with the c.536A > G (p.Y179C) MAP pathogenic variant in the *MUTYH* gene [6, 17, 18]. The splice site alteration is not reported in either COSMIC (http://cancer.sanger.ac.uk/cancergenome/projects/cosmic/) or dbSNP (http://www.ncbi.nlm.nih.gov/projects/SNP/) databases. In three FAP probands no pathogenic mutation was found in *APC* and *MUTYH*; one proband (FAP5) of these three showed the *MSH6* variant c.663A > C (p.E221D; rs41557217), which is described as HNPCC-related of uncertain pathogenicity in NCBI’s ClinVar database (http://www.ncbi.nlm.nih.gov/clinvar/) [19].

Among the 8 HNPCC probands, pathogenic variants were detected in 7: three in *MLH1* and four in *MSH2* [20–24]. Of interest, in proband HNPCC1 a *MLH1* gene mutation was associated with the c.998C > T (p.T333I) variant in the *MSH6* gene, which is classified as of uncertain pathogenicity in NCBI’s ClinVar database [19].

Known non-pathogenic polymorphisms found were as follows (Fig. 1). The *APC* c.5465 T > A (p.V1822D) variant, which has an allelic frequency of 82 % in Caucasian population [25], in 5 FAP probands (FAP 2,4,5,7,8) and 4 HNPCC probands (HNPCC 1,5,6,8). The *MUTYH* c.1014G > C (p.Q338H) [26] in 3 FAP probands (FAP 1,6,8) and 2 HNPCC probands (HNPCC 3,7). The *MLH1* c.655A > G (p.I219V) variant was identified in 5 FAP probands (FAP 1,3,5,6,7) and 2 HNPCC probands (HNPCC 4,7). All the above variants were also detected at Sanger sequencing.

### Targeted NGS has blind spots

In the present series of 16 probands, all mutations detected at NGS were also found at Sanger sequencing (Table 1). However, the HNPCC7 proband c.840_841delAT (p.L280Ffs*3) mutation in the *MSH2* gene was identified only at Sanger sequencing. This mutation is located in a region rich of homopolymer stretches, which renders it both difficult to amplify and prone to artifacts. As a result, that region had virtually no coverage (i.e. it was covered around 20x but the base and mapping qualities were not sufficient for variant calling). A total of 2225 pathogenic variants are described in the COSMIC database for *APC*, *MUTYH*, *MLH1*, *MSH2*, *MSH6* (Table 2). Of these variants, our targeted NGS custom panel shows a clear sequence of the DNA regions that harbor 2108 (94.7 %) of these variants, that would then be automatically identified by the Variant Caller Plugin software (Torrent Suite Software v3.6; Life Technologies), while the regions harboring the remaining 117 variants present problems. Twenty-seven (1.2 %) variants are masked at the software (Additional file 2: Table S2), i.e. these variants are automatically identified but their proximity to homopolymer stretches causes imprecise calls that require visual inspection of the region, using the Integrative Genomics Viewer (IGV) v2.2 (Broad Institute), to be correctly identified. In particular, COSMIC variants that are masked and require visual inspection to be sorted out are 12 for *APC*; 1 for *MUTYH*; 4 for *MLH1*; 3 for *MSH2*; 7 for *MSH6* (Additional file 2: Table S2). The remaining 90 variants (4.0 %) are blind spots, i.e. these variants are located at the end of an amplicon or within homopolymer stretches; in these cases neither the software nor visual inspection are able to discern between an artifact and a true alteration. In particular, the blind spot COSMIC variants are 51 for *APC*; 10 for *MUTYH*; 15 for *MLH1*; 5 for *MSH2*; 9 for *MSH6* (Additional file 2: Table S2).

**Table 2:** Mutation detection with Next-Generation and Sanger sequencing

GENE	Coding region in bp	COSMIC mutations^a^	N. Sanger needed		Next-Generation Sequencing			
				Solved	Solved by IGV^b^	Sensi tivity	Blind Spots^c^	N. Sanger to solve blind spots
*APC*	8538	1670	55	1607	12	96.9 %	51	11
*MUTYH*	1854	40	16	29	1	75.0 %	10	2
*MHL1*	2524	163	39	144	4	90.8 %	15	6
*MSH2*	11,227	152	30	144	3	96.7 %	5	4
*MSH6*	4080	200	36	184	7	95.5 %	9	4
*Total*	28,223	2225	176	2108	27	96.0 %	90	27

^a^ Mutations listed in COSMIC database (http://cancer.sanger.ac.uk/cancergenome/projects/cosmic/)

^b^ Integrative Genomics Viewer (IGV v.2.1, Broad Institute) software

^c^ Mutations within homopolymer stretches or artifact-prone regions of the genes

### Targeted NGS blind spots are solved at Sanger sequencing

The analysis of the entire coding sequence for the *APC*; *MUTYH*; *MLH1; MSH2* and *MSH6* genes using Sanger sequencing requires a number of reactions summing up to 55 for *APC*, 16 for *MUTYH*, 39 for *MLH1*, 30 for *MSH2* and 36 for *MSH6*. Applying our NGS panel the number of Sanger sequencing reactions to explore the blind spots would require a reduced number of reactions: 11 for *APC*, 2 for *MUTYH*, 6 for *MLH1*, 4 for *MSH2* and 4 for *MSH6* (Table 2).

### Cost and time comparison

Cost and time comparison between NGS and Sanger sequencing are summarized in Table 3. The cost of consumables for any single PCR product analysis by Sanger sequencing was €28.0 [27]. For Ion Torrent sequencing, our initial loading of 4 samples per 318 chip was far beyond our theoretical needs, this permitting to assess the performances of a totally new panel while being sure to get results even in the worst scenario. In a routine setup, considering that even a sample (HNPCC6) producing only 177,000 reads had an average coverage of 670X with all non-blinded regions covered >20X, the maximum number of samples chargeable on a 318 chip (max 6,000,000 total reads) for Ion PGM sequencing is 30, significantly reducing the overall costs to €325.0 per sample.

**Table 3:** Comparison of indicative costs and time per sample

	FAP		HNPCC	
	*Sanger*	*NGS + Sanger*	*Sanger*	*NGS + Sanger*
PCR reactions	71 reactions × 28.0 € = 1988.0 €	13 reactions × 28.0 € = 364.0 €	105 reactions × 28.0 € = 2940.0 €	14 reactions × 28.0 € = 392.0 €
PGM^a^	0	325.0 €	0	325.0 €
Total costs	1988.0 €	689.0 €	2940.0 €	717.0 €
Indicative timing	25 days	15 days	28 days	16 days

^a^ PGM, Ion Torrent Personal Genome Machine (Life technologies)

Grouping cost analysis for FAP (*APC* and *MUTYH* genes) and HNPCC (*MLH1*, *MSH2* and *MSH6* genes) syndromes, NGS analysis integrated by Sanger sequencing results significantly cheaper in comparison to Sanger sequencing alone for both types of probands (Table 3). As expected, the mean turn-around-time for NGS-based analysis were significantly lower in comparison to conventional Sanger sequencing.

## Discussion

The last few years have been characterized by considerable consolidation of our genetic understanding of hereditary CRC syndromes, leading to an increasing request for genetic testing [28, 29]. However, the costs and time required for the analysis of multiple genes using Sanger sequencing is limiting a wider application of genetic testing. Next-generation sequencing approaches permit the simultaneous analysis of multiple genes in a limited period of time. This multigene diagnostic approach has been already fruitfully applied in oncology [30, 15], and its introduction in routine practice for the molecular characterization of probands of colorectal cancer syndromes is foreseen [31].

In this study we compared the gold standard Sanger sequencing to the Ion Torrent NGS approach for diagnostic application in the screening of familial CRC. A series of 16 probands were investigated for germline intragenic mutations in five CRC familial syndromes-associated genes (*APC*, *MUTYH*, *MLH1*, *MSH2*, *MSH6*).

The NGS approach used herein and Sanger sequencing gave overlapping results. Thirteen of 14 pathogenic variants in the genes tested were detected by both technologies. Only one *MSH2* pathogenic mutation (p.L280Ffs*3) was identified by Sanger sequencing but not by the NGS. This is due to a limitation of NGS in resolving sequences corresponding to DNA homopolymeric stretches. On the other hand, the multiplex NGS approach has the advantage residing in the possibility to analyze multiple genes in multiple samples simultaneously, thus reducing costs and turnaround time in comparison to Sanger sequencing. With our custom panel, only three days for library construction and sequencing of 8 cases was requested; the library production is quicker as multiplex PCR reactions happen in only one/two tubes, requiring less DNA and hands-on time even in absence of automation; the sequencing and analysis procedure may be carried on overnight reducing waiting times; the visual analysis of NGS tracks is faster and easier than the verification of electrophoretic peaks on conventional Sanger sequencing.

To evaluate the performance of our NGS custom panel we assessed its capability to resolve the DNA sequences corresponding to the 2225 pathogenic variants reported in the COSMIC database for *APC*, *MUTYH*, *MLH1*, *MSH2*, *MSH6*. The analysis using the Torrent Suite Software clearly resolves the DNA sequences where 2108 (94.7 %) of these variants occur. The remaining 117 mutations listed in COSMIC reside inside or in close proximity to homopolymer stretches, and this causes problems in the sequencing reaction of these areas as well as imprecise calls by the software. Of these 117 regions, 27 (1.2 %) are automatically identified by the software but without a clear call, which can be correctly resolved by the visual inspection of the region; this visual inspection is however routinely performed for all called variants and as such already part of analysis times depicted in Table 3. The remaining 90 variants (4.0 %) are blind spots, i.e. these variants are located at the end of an amplicon or within homopolymer stretches, and in these cases neither the software nor visual inspection are able to discern between an artifact and a true alteration. In summary, our custom panel would miss 4 % (90/2225) of pathogenic variants that would need a small set of Sanger sequencing reactions to be solved. Moreover, longer amplicon (375 bp) designs have been made available, and such constant improvement in software design, together with the continuous engineering of reagents (improved sequencing polymerases have become available) is also expected to solve most of the blind spots, reducing the need of complementary Sanger sequencing. Another by-design limitation of the present AmpliSeq panel is that it cannot detect large (>100 bp) insertion and deletions, due to the size (100–200 bp) of the amplicons produced by multiplex PCR. These large insertions and deletion are anticipated to be detectable by a copy number variation approach that is available in the latest version of both the AmpliSeq designer and analysis software.

An important advantage of NGS resides in the possibility to analyze genes that are usually not assessed due to adjunctive costs not covered by the National Health Systems, and this may uncover previously unknown combined mutations in affected families or individuals. Probands FAP5 and FAP7 are representative examples of the benefit of using a multigene mutational analysis. In FAP7 Sanger sequencing identified only an *APC* c.3920 T > A (p.I1307K) mutation, and this would have probably stopped the analysis for this patient, as it is a FAP pathogenic mutation, albeit its clinical significance is still controversial [32, 33]; the NGS multiplex approach revealed a coexistent *MUTYH* c.536A > G (p.Y179C) mutation, which is reported as pathogenic and related to MAP syndrome [6, 17, 18]. Similarly, in FAP5 the presence of *MSH6* c.663A > C (p.E221D) was identified, this variant has been related to Lynch syndrome although it is of uncertain clinical significance [19].

## Conclusions

Despite the limitation of hard sequencing regions, the multigene and multi-sample NGS approach showed major benefits on costs and time required compared to conventional Sanger sequencing. Therefore, NGS technology can be included as an adequate diagnostic method for the identification of intragenic mutation testing of familial CRC syndromes, complemented in the mutation-negative cases with a reduced number of Sanger sequences to resolve the DNA regions not adequately assessed by NGS.

## Additional files

Additional file 1: Table S1. NGS custom panel targeted regions. (DOCX 28 kb)
Additional file 2: Table S2. Masked and Blind Spot in Custom Panel. (DOCX 17 kb)

## Abbreviations

CRC: Colorectal cancer
FAP: Familial adenomatous polyposis
HNPCC: Hereditary nonpolyposis colorectal cancer
MAP: *MUTYH*-associated polyposis
NGS: Next-generation sequencing
