# Supplementary material for: Next-generation sequencing for genetic testing of familial colorectal cancer syndromes
Source: Hered Cancer Clin Pract. 2015 Aug 21;13:18. doi: 10.1186/s13053-015-0039-9 (PMC4546256; doi:10.1186/s13053-015-0039-9)
Supplement: Additional file 1: Table S1. — NGS custom panel targeted regions. (DOCX 28 kb) [file 13053_2015_39_MOESM1_ESM.docx]

**Additional file 1: Table S1.** NGS custom panel targeted regions.

chr1 45794956 45795135 MUTYH

chr1 45796079 45796294 MUTYH

chr1 45796827 45797036 MUTYH

chr1 45797046 45797273 MUTYH

chr1 45797145 45797348 MUTYH

chr1 45797348 45797563 MUTYH

chr1 45797593 45797825 MUTYH

chr1 45797799 45798030 MUTYH

chr1 45798008 45798238 MUTYH

chr1 45798171 45798410 MUTYH

chr1 45798356 45798583 MUTYH

chr1 45798504 45798711 MUTYH

chr1 45798714 45798933 MUTYH

chr1 45798879 45799092 MUTYH

chr1 45798962 45799110 MUTYH

chr1 45799111 45799311 MUTYH

chr1 45800008 45800229 MUTYH

chr1 45805804 45806029 MUTYH

chr2 47630285 47630450 MSH2

chr2 47630445 47630595 MSH2

chr2 47635515 47635602 MSH2

chr2 47635560 47635635 MSH2

chr2 47635636 47635784 MSH2

chr2 47637172 47637272 MSH2

chr2 47637251 47637338 MSH2

chr2 47637312 47637523 MSH2

chr2 47639524 47639682 MSH2

chr2 47639678 47639810 MSH2

chr2 47641290 47641512 MSH2

chr2 47641513 47641678 MSH2

chr2 47643387 47643605 MSH2

chr2 47656824 47656934 MSH2

chr2 47656935 47657136 MSH2

chr2 47672608 47672730 MSH2

chr2 47672731 47672902 MSH2

chr2 47690062 47690163 MSH2

chr2 47690163 47690234 MSH2

chr2 47690200 47690412 MSH2

chr2 47693767 47693936 MSH2

chr2 47693813 47693984 MSH2

chr2 47698057 47698272 MSH2

chr2 47702142 47702215 MSH2

chr2 47702197 47702271 MSH2

chr2 47702256 47702336 MSH2

chr2 47702337 47702509 MSH2

chr2 47703350 47703525 MSH2

chr2 47703526 47703743 MSH2

chr2 47705349 47705469 MSH2

chr2 47705470 47705686 MSH2

chr2 47707773 47707895 MSH2

chr2 47707896 47708056 MSH2

chr2 47709869 47709961 MSH2

chr2 47709962 47710161 MSH2

chr2 48010327 48010432 MSH6

chr2 48010430 48010654 MSH6

chr2 48017979 48018156 MSH6

chr2 48018157 48018319 MSH6

chr2 48023022 48023104 MSH6

chr2 48023053 48023133 MSH6

chr2 48023111 48023222 MSH6

chr2 48025715 48025790 MSH6

chr2 48025752 48025911 MSH6

chr2 48025912 48026117 MSH6

chr2 48026118 48026333 MSH6

chr2 48026334 48026558 MSH6

chr2 48026559 48026779 MSH6

chr2 48026780 48026984 MSH6

chr2 48026983 48027118 MSH6

chr2 48027119 48027314 MSH6

chr2 48027315 48027533 MSH6

chr2 48027533 48027730 MSH6

chr2 48027731 48027868 MSH6

chr2 48027869 48028088 MSH6

chr2 48028089 48028312 MSH6

chr2 48030524 48030749 MSH6

chr2 48030750 48030880 MSH6

chr2 48031994 48032178 MSH6

chr2 48032697 48032825 MSH6

chr2 48032826 48032939 MSH6

chr2 48033197 48033360 MSH6

chr2 48033361 48033575 MSH6

chr2 48033500 48033652 MSH6

chr2 48033653 48033844 MSH6

chr2 48033877 48034033 MSH6

chr3 37034975 37035211 MLH1

chr3 37038045 37038268 MLH1

chr3 37042388 37042503 MLH1

chr3 37042504 37042574 MLH1

chr3 37045813 37046038 MLH1

chr3 37048441 37048565 MLH1

chr3 37050238 37050463 MLH1

chr3 37053227 37053436 MLH1

chr3 37053443 37053661 MLH1

chr3 37055860 37056082 MLH1

chr3 37058942 37059156 MLH1

chr3 37061763 37061995 MLH1

chr3 37066987 37067181 MLH1

chr3 37067182 37067334 MLH1

chr3 37067335 37067508 MLH1

chr3 37070136 37070274 MLH1

chr3 37070275 37070471 MLH1

chr3 37081619 37081823 MLH1

chr3 37083668 37083861 MLH1

chr3 37088983 37089208 MLH1

chr3 37089946 37090164 MLH1

chr3 37090342 37090560 MLH1

chr3 37091932 37092156 MLH1

chr5 112043365 112043596 APC

chr5 112090416 112090608 APC

chr5 112090609 112090764 APC

chr5 112101930 112102152 APC

chr5 112102725 112102919 APC

chr5 112102920 112103106 APC

chr5 112111245 112111355 APC

chr5 112111356 112111547 APC

chr5 112116385 112116518 APC

chr5 112116519 112116694 APC

chr5 112128061 112128166 APC

chr5 112128167 112128263 APC

chr5 112136793 112136969 APC

chr5 112136970 112137128 APC

chr5 112151119 112151321 APC

chr5 112154619 112154842 APC

chr5 112154839 112155052 APC

chr5 112157378 112157604 APC

chr5 112157605 112157791 APC

chr5 112162766 112162878 APC

chr5 112162879 112162955 APC

chr5 112163599 112163777 APC

chr5 112164500 112164570 APC

chr5 112164566 112164647 APC

chr5 112164595 112164728 APC

chr5 112170614 112170751 APC

chr5 112170752 112170839 APC

chr5 112170820 112170892 APC

chr5 112173217 112173425 APC

chr5 112173426 112173649 APC

chr5 112173650 112173800 APC

chr5 112173801 112173994 APC

chr5 112173995 112174215 APC

chr5 112174216 112174408 APC

chr5 112174409 112174543 APC

chr5 112174544 112174735 APC

chr5 112174736 112174936 APC

chr5 112174937 112175158 APC

chr5 112175159 112175284 APC

chr5 112175285 112175453 APC

chr5 112175454 112175682 APC

chr5 112175683 112175875 APC

chr5 112175876 112176090 APC

chr5 112176091 112176184 APC

chr5 112176185 112176378 APC

chr5 112176379 112176588 APC

chr5 112176589 112176808 APC

chr5 112176809 112176985 APC

chr5 112176986 112177196 APC

chr5 112177197 112177415 APC

chr5 112177416 112177585 APC

chr5 112177586 112177794 APC

chr5 112177795 112177988 APC

chr5 112177989 112178193 APC

chr5 112178193 112178415 APC

chr5 112178416 112178614 APC

chr5 112178615 112178838 APC

chr5 112178839 112179059 APC

chr5 112179060 112179279 APC

chr5 112179280 112179460 APC

chr5 112179461 112179544 APC

chr5 112179545 112179752 APC

chr5 112179753 112179933 APC
