# Supplementary material for: Next-generation sequencing for genetic testing of familial colorectal cancer syndromes
Source: Hered Cancer Clin Pract. 2015 Aug 21;13:18. doi: 10.1186/s13053-015-0039-9 (PMC4546256; doi:10.1186/s13053-015-0039-9)
Supplement: Additional file 2: Table S2. — Masked and Blind Spot in Custom Panel. (DOCX 17 kb) [file 13053_2015_39_MOESM2_ESM.docx]

**Additional file 2: Table S2.** Masked and Blind Spot in Custom Panel.

| GENE | MASKED | BLINDED |
| --- | --- | --- |
| *APC* | COSM19131 COSM304578 COSM1432187 COSM201299 COSM99775 COSM18988 COSM18785 COSM29335 COSM1432424 COSM33466 COSM1432443 COSM1541288 | COSM298221 COSM288426 COSM1432197 COSM1432206 COSM201301 COSM18851 COSM19062 COSM293922 COSM18724 COSM290300 COSM19262 COSM19073 COSM23583 COSM19046 COSM26697 COSM18809 COSM99780 COSM23588 COSM19601 COSM1180986 COSM19713 COSM24940 COSM328648 COSM1645209 COSM29576 COSM19598 COSM328655 COSM19623 COSM32442 COSM23597 COSM19054 COSM19299 COSM1432425 COSM23600 COSM19697 COSM98436 COSM18940 COSM86020 COSM19695 COSM19000 COSM18734 COSM41621 COSM19020 COSM18561 COSM18576 COSM18875 COSM18874 COSM18871 COSM18882 COSM33687 COSM18816 |
| *MUTYH* | COSM535484 | COSM1343046 COSM347965 COSM910160 COSM1639902 COSM1195469 COSM1645292 COSM253773 COSM1195175 COSM426362 COSM175172 |
| *MLH1* | COSM382275 COSM94707 COSM26081 COSM25890 | COSM1422578 COSM1422582 COSM1422583 COSM1422589 COSM1422596 COSM1617522  COSM1422563 COSM330644 COSM41604 COSM1043965 COSM269591 COSM96029 COSM420033 COSM1422564 COSM1043966 |
| *MSH2* | COSM327050 COSM26126 COSM1286279 | COSM26120 COSM1408250 COSM26124 COSM26123  COSM330654 |
| *MSH6* | COSM1408291 COSM13383 COSM13398 COSM13384 COSM29732 COSM442968 COSM393571 | COSM1408295 COSM1408296 COSM1239478 COSM308681 COSM330655 COSM1408313  COSM35856 COSM35885 COSM1327059 |
